# Hemolytic Uremic syndrome associated with pregnancy: Outcome from acute Kidney Injury

**DOI:** 10.12669/pjms.36.6.2931

**Published:** 2020

**Authors:** Rubina Naqvi

**Affiliations:** 1 Prof. Dr. Rubina Naqvi Department of Nephrology, Sindh Institute of Urology and Transplantation, Karachi, Pakistan

**Keywords:** AKI, RIFLE criteria, Hemolytic Uremic Syndrome, Pregnancy, Plasmapheresis

## Abstract

**Objective:**

To report here, case series of women developing acute kidney injury (AKI) in association with hemolytic uremic syndrome (HUS) during pregnancy or within days postpartum.

**Methods:**

Subjects for the study reported here comprised of a cohort of 49 women referred from OBGYN and diagnosed having AKI and HUS. AKI was defined according to RIFLE criteria and HUS on basis of hematological, biochemical and histological features. All patients had normal size kidneys on ultrasonography and no previous co- morbidity.

**Results:**

From January 2000 – January 2020, 49 such women were admitted/ referred to this facility. The mean age of these patients was 29.02±5.258 years. Two had HUS during pregnancy while rest during postpartum. Majority of women had more than one insults these include hemorrhages, intrauterine deaths, operative measure (lower segment caesarean section). Renal replacement was required in all women. Complete renal recovery was observed in 14 patients, while one died during acute phase of illness. CKD-V developed in 17 patients, 16 patients lost long term follow up, but were dialysis free till last follow up and one left against medical advice during acute phase of illness. Treatment with plasmapheresis revealed significantly better renal recovery (p value 0.03) in this group of patients.

**Conclusion:**

AKI with background of Hemolytic Uremic Syndrome (HUS) may remain irreversible in many of these young women. Plasmapheresis should be offered to patients with established diagnosis of HUS.

## INTRODUCTION

Early theories in pathogenesis of HUS proposed occlusion of arterioles with fibrin, causing mechanical destruction of platelets and red blood cells while passing through these occluded arterioles. Abnormal plasma factors have also been postulated as the cause of intra vascular platelet activation in HUS.^1^ Further down, atypical hemolytic uremic syndrome during pregnancy or soon after child birth (PaHUS) is reported to be often associated with coagulation abnormalities with disseminated intra vascular coagulation in 30% of cases. Verotoxin producing E. Coli infection was also supposed to be one of possibility triggering PaHUS.^2^ With further advancement in research it was stated that uncontrolled complement activation during pregnancy can lead to thrombotic microangiopathy and PaHUS.^3^

The incidence of PaHUS has been reported 1 in 25,000 pregnancies and accounts for 20% of all HUS cases in women.^4^,^5^ We have previously reported 30.47% of total HUS was associated with pregnancy.^6^ Another study from country reported Pregnancy related AKI found HUS in 6.3% women.^7^

Risks of different forms of thrombotic microangiopathies including ADAMTS 13 deficiency associated HUS increase during pregnancy.^8^ During normal pregnancy complement activation occurs at site of placental attachment to uterine wall and alternate pathway regulation depends on CD 59 and Decay accelerating factor.^9^ PaHUS is a devastating disease where clinical understanding and treatment options are still limited.^10^ It may develop as a complication of pre- eclamsia^11^ in association with HELLP syndrome or sometimes with extensive hemorrhage pre or post partum.^12^ Progressing to life threatening variant of preeclampsia with severe microangiopathic hemolytic anemia and thrombocytopenia along with AKI and liver damage in some women. In this situation coagulation may be found to be abnormal with disseminated intravascular coagulation.^13^ We aim to report our experience of PaHUS from a tertiary renal care center by giving their presentation, management and outcome.

## METHODS

In this retrospective study we reviewed clinical records of all women who were brought to Sindh Institute of Urology and Transplantation (SIUT) from January 2000 – January 2020 and in whom the diagnosis of AKI and HUS during pregnancy or in post partum period was established.

In this study AKI was defined according to RIFLE criteria; and all patients fall in one of category between ‘R’ and ‘L’, though patients referred here in ‘R’ category are very few and were mainly from OBGYN department of same hospital (Civil Hospital, Karachi).

**Fig.1:**
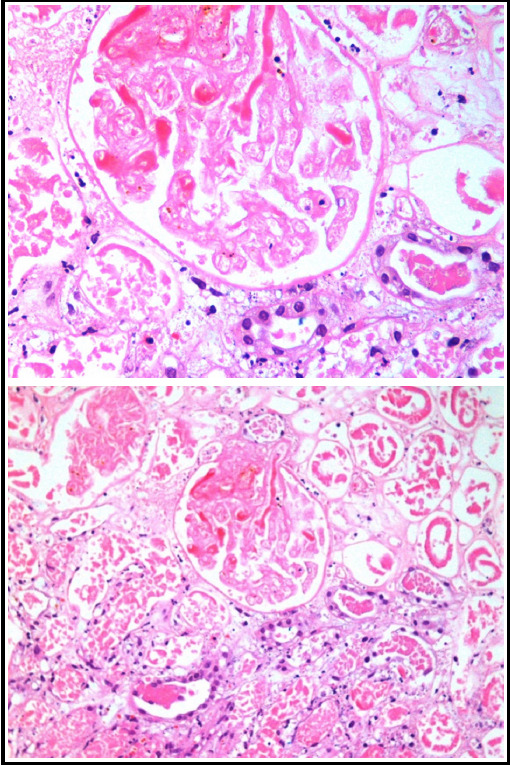
Histological findings in two different magnifications.

As previously described;^6^ HUS was defined as association of following criteria: mechanical hemolytic anemia (hemoglobin <11 g/dl, raised lactate dehydrogenase, presence of schistocytes or fragmented red blood cells on blood peripheral film) and thrombocytopenia (platelet count <150x10^3^/μl). All patients had renal biopsy done and diagnosis of HUS was confirmed on biopsy when met the criteria of fragmented RBC, extravasations of red blood cells, obliteration of capillary lumina and presence of fibrin thrombi. (Fig.1) Patient demographics were recorded for laboratory parameters of CBC, renal chemistry, liver function tests, coagulation profile, urinalysis, serum LDH and complement levels. Factor H; which is done by ELISA method in our laboratory was available for patients who were registered after March 2015. Management of all patients was recorded for need for renal replacement therapy (RRT), plasmapheresis (PP), its duration and finally outcome. Only data from patients fulfilling criteria of definitions for AKI and HUS, be it hematological/ biochemical or histological, were included in the study.

High-power view showing an infarcted glomerulus with dark red colored fibrin thrombi in some glomerular capillaries. A few tubules are still viable in the lower part of the field. (H&E, ×400).

Medium-power view showing an infarcted glomerulus with fibrin thrombi in some glomerular capillaries. A few tubules are still viable in the lower part of the field. (H&E, ×200).

All patients were followed up till death, complete renal recovery or no recovery (labeled as CKD-V). Patients with partial recovery of kidney function and who lost to follow up were considered among the partial recovery of patients. Patients with HELLP syndrome or other identifiable cause of secondary HUS, like lupus or antiphospholipid syndrome were excluded from study.

Informed consent for starting RRT, doing biopsy, proceeding with PP or sharing information without personal identification was achieved from all women or their designated family member. Verbal consent from ERC of institution was taken as data was retrieved from records documented process was not suggested by ERC.

### Statistical Methods

Statistical analysis was done on SPSS version 20.0 (IBM, USA). Mean ± standard deviation was expressed or median with inter-quartile range provided for continuous variables. Frequencies and percentages computed for categorical variables. The chi square test of independence was used to determine the proportional difference between outcomes with different presenting symptoms or outcome with provision of plasmapheresis. P value <0.05 was considered as significant.

## RESULTS

During the studied period from January 2000 - January 2020, total 49 women were diagnosed to have AKI with Pa HUS. Basic demographics of studied population and initial laboratory parameters are given in Table-I.

**Table-I:** Baseline demographics and laboratory values.

Parameters	Mean ± SD	Median	Inter Quartile Range
Age (years)	29.02±5.258	29	25-33
Duration of Insult (days)	8.39±9.673	6	2.5-10
Hemoglobin (11.5-15.4G/dl)	7.516±1.710	6.2	6.2- 8.9
Total leukocyte count (4-11 x10^3^/μl)	19.58±9.252	9.5	12.55-24.50
Platelet (150-400 x10^3^/μl)	152.08±122.62	70	70-209.50
Urea (15-39mg/dl)	166.96±75.219	119	117-209
Creatinine (mg/dl) (0.5-1.5)	8.734±4.027	7.790	5.7-10.8
Sodium (136-156 meq/l)	134.71±7.789	136	132-138
Potassium (3.5-5.1 meq/l)	4.837± 0.977	4.6	4.15- 5.35
LDH (<247 U/L)	2421.92±1190.239	2208	1435-3273
Total Bilirubin (0.2-1.0 mg/dl)	2.06±3.622	0.7	0.56 -1.43
AST (10-42 U/L)	145.61±226.296	55	29-180
ALT (10-40 U/L)	105.49±144.760	49	20-141.50
Factor H(250-600 mcg/ml)	425.93±333.188	291	111.75 -762.25
C3 (0.79-1.52 g/L)	1.051±0.990	0.99	0.805-1.235

LDH= Lactate dehydrogenase, AST= Aspartate Aminotransferase,

ALT = Alanine Aminotransferase, C3= complement factor

Majority had more than one insult as contributing factors for example ante-partum hemorrhage (APH) as single factor was only found in one patient while in association with intra uterine death (IUD) of fetus in seven, APH, IUD with childbirth through lower segment caesarean section (LSCS) in 11, APH, IUD with postpartum hemorrhage (PPH) in three, APH, IUD and PPH in three, APH with LSCS in two, IUD and LSCS in 2, IUD and PPH in two, LSCS with twin pregnancy in one patient and only LSCS in 5 women. Two patients developed hemeparesis after child birth and during course of illness in hospital. Two of 49 developed HUS during pregnancy while rest was post partum. Time from first day of symptom to reaching to our hospital was recorded as days of insult and given in Table-I.

Only one patient had non oliguric AKI, while 16 (32.7%) had oliguria and 32 (65.3%) were anuric on presentation. Renal replacement therapy (which was done in form of hemodialysis) was required in all patients.

The urinalysis during hospital stay was possible in 37 (75.5%) women, rest had absolute anuria. Among these all had microscopic hematuria and 1-4+protein on dipstick. Deranged International Normalized Ratio (INR) was found in only one patient at time of admission. Renal biopsy was performed in 48 of 49 patients, in 21 biopsies along with fragmentation and extravasation of red blood cells, obliteration of capillary lumina and presence of fibrin thrombi it revealed focal cortical necrosis, diffuse cortical necrosis in one and acute tubular necrosis in three. One biopsy along with fibrin thrombi revealed some chronic sclerosing changes in glomeruli.

One of 49 (2%) patient died during acute phase of illness, one left against medical advice when explained about plasmapheresis and expected outcome, 14 (28.6%) got complete renal recovery, 17 (34.7%) remained dialysis dependent beyond 90 days and 16 (32.7%) were patients who were dialysis free but not achieved normal renal functions yet and lost long term follow-up. Among those who recovered 6- 13 hemodialysis sessions were required. Plasmapheresis (PP) was not offered to 12 patients of these two were the women registered during very initial part of study, some refused for the therapy by family and for some reason was not clear from records. Later an average of 14 sessions of PP done in all and 22 sessions in one who developed HUS during pregnancy, though she recovered normal renal functions after initial phase of therapy but PP continued fortnightly till four weeks postpartum.

While other factors remained statistically insignificant to renal outcome it was found that provision of PP was found statistically significant and useful in renal recovery. (Table-II)

**Table-II:** Predictors for Outcome.

Predictor	P value
Oliguria	0.605
Anuria	0.163
Days of Insult	0.939
Mean of Platelet count on presentation	0.739
Mean of Urea on presentation	0.744
Mean of serum creatinine on presentation	0.752
Plasmapheresis done	0.03

*chi sq test of independence.

## DISCUSSION

This is the largest to date study from our country assessing the presentation, management and outcome in PaHUS. Previously we have published a series of AKI secondary to HUS which included all causes; we have not performed complement genetic analysis on these patients so cannot comment on the fact whether PaHUS and other causes HUS differ in this regard.^6^ But others have reported no significant association of complement genetic make with PaHUS.^10^ The targeted inhibition of the complement alternative pathway has been worked out and some promising results have been described in some studies.^4^,^12^,^14^ The use of anti C5 monoclonal antibody, eculizumab has been published in reflecting its results in both PaHUS and other causes HUS, some have shown successful treatment with use of eculizumab with normalization of all hematologic features and rapid renal recovery.^10^ But main hindrance in use of this monoclonal antibody is its cost and non availability in many countries like ours. Many studies done before trials of eculizumab have shown PP as effective treatment in removing overactive proteins and functionally defective proteins.^15^-^17^

Plasma exchange has been suggested first line therapy for typical HUS associated with severe renal insufficiency or brain impairment for last many years and early plasmapheresis, with or without eculizumab, was supposed to remain the cornerstone of treatment.^18^ We have seen significantly good prognosis in terms of renal recovery in all causes of HUS and in present PaHUS.^6^ An interesting finding in our experience of AKI secondary to HUS was that during initial years of study we mostly use to see HUS with other causes and except for two rest of cases in present study were brought here during last four years, reason for this cannot be explained on scientific grounds. All histological samples were analyzed at our own histopathology department by same team members thus question of this variation is waived here.

Previously a Spanish study has shown an association of LSCS with PaHUS,^10^ in present series 22 of 49 (45%) women has had LSCS done, in 5 of these 22 LSCS was only insult while in others it was associated with one or more than one other factor described above in results.

In present study which is analysis of retrospective data, some women were not considered for plasmapheresis (especially during early part of study), reason for which is not clear in records but it was evident that those who were offered this therapy revealed statistically significant better renal outcome (p value 0.03).

In a study in 2014 from Rawalpindi, Pakistan, among pregnancy associated AKI 13/88(15%) had HUS, however their outcome was not mentioned.^19^ In present study we have seen complete renal recovery in 28.6% and irreversible renal failure in 34.7% women.

From same institution we have previously published large series of pregnancy associated AKI where over a span of 25 years 1,441 such women were treated but there HUS and HELLP were described under one category among many others.^20^ Another study from country reported 111 patients with pregnancy associated AKI, of these seven were diagnosed with HUS (3 had concomitant preeclampsia). However, no further data related to morbidity and mortality was mentioned.^7^

### Limitations of study

Genetic screening and testing for autoantibodies were not performed in these patients. Monoclonal antibody Eculizumab was not used in any of these patients as it is not available in country.

## CONCLUSION

Despite limitations with current study it is evident from data that prognosis in terms of renal recovery is not very good in AKI and HUS in obstetrical scenario where all patients are young. It is important to know the burden of disease so that future diagnostics and treatment modalities can be planned. PP should be offered to all women developing PaHUS especially when Eculizumab is not available or affordable.
